# Clinico-pathological Spectrum of Oral Cavity Lesions at a Tertiary Care Center in Central Nepal: A Descriptive Cross-sectional Study

**DOI:** 10.31729/jnma.5539

**Published:** 2021-02-28

**Authors:** Shankar Bastakoti, Gambhir Shrestha, Dej Kumar Gautam, Ishan Dhungana, Nandita Jha, Greta Pandey, Suraj Upreti, Ashmita Shrestha, Ranjan Raj Bhatta

**Affiliations:** 1 Department of Pathology, BP Koirala Memorial Cancer Hospital; 2 Department of Cancer Prevention, Control and Research, BP Koirala Memorial Cancer Hospital; 3 Department of Surgical Oncology, ENT, Head and Neck Unit, BP Koirala Memorial Cancer Hospital; 4 Department of Pathology, Universal College of Medical Sciences

**Keywords:** *oral cancer*, *squamous cell carcinoma*, *tobacco*

## Abstract

**Introduction:**

Head and neck cancer is the sixth most common cancer in the world. The disease burden is increasing at an alarming rate in developing Southeast Asian countries. This study aims to report the histopathological spectrum of oral cavity lesions at a tertiary cancer center in central Nepal.

**Methods:**

This study included all those cases of oral cavity lesions, of which diagnostic biopsy was done from January 2018 to December 2019. The data were retrieved from the Department of Pathology of BP Koirala Memorial Cancer Hospital. The study proposal was approved by the Institutional Review Committee at BPKMCH (Ref: 247/2020) on 28th June 2020.

**Results:**

A total of 851 cases of oral cavity lesions were included in this study. The mean age of the study population was 55.9 years, with male to female ratio of 3:1. Malignant lesions composed of 472 (55.5%) cases followed by premalignant lesion of 104 (12%). More than 453 (95%) malignant cases were squamous cell carcinoma, of which 342 (75%) were a well-differentiated type. The buccal cavity is the most common site of malignant lesion 212 (45%), followed by tongue 96 (20%) and lower gingivobuccal region 86 (18%).

**Conclusions:**

Malignant lesions are the most common histopathological findings in the oral cavity lesion with squamous cell carcinoma type. Oral cancer is common cancer that can be prevented and cured if detected early.

## INTRODUCTION

Cancer is currently one of the major global issues. As per World Health Report 2018, the disease burden has risen to 18.1 million new cases and 9.6 million deaths in 2018. One in 5 men and one in 6 women worldwide develop cancer during their lifetime.^1^ Head, and neck cancer is the sixth most common cancer in the world. The disease burden is increasing at an alarming rate in developing Southeast Asian countries. As Globacon 2018 data, the most common cancer in males is oral cavity cancer (OCC) in South-East Asia, surpassing the previously stated lung cancer. The incidence rate is 7.4% in 100,000 population, with a mortality rate accounting for 6.7% in 100,000 population.^2^

The majority of the cancers that occur in the oral cavity are oral squamous cell carcinomas (OSCC) arising from the squamous epithelial lining of buccal mucosa, tongue, the floor of mouth, palate, and lip.^3^

This study aims to present the clinicopathological spectrum of oral cavity lesions in a tertiary cancer hospital in Nepal.

## METHODS

This study included all those cases of Oral cavity lesions, of which diagnostic biopsy was done in BP Koirala Memorial Cancer Hospital (BPKMCH), Bharatpur, Chitwan, Nepal, from January 2018 to December 2019. BPKMCH is the largest comprehensive cancer center in Nepal, with most new cancer cases treated. The data were retrieved from the Department of Pathology of BPKMCH. Those cases with non-diagnostic biopsy interpretations slides were received for review only, and incomplete information was excluded.


$$\begin{array}{ll} \text{n} & ={\text{Z}}^{\text{2}}\times \text{p}\times \text{q}/{\text{e}}^{\text{2}}\\ & ={\left(1.96\right)}^{2}\times (0.5)\times (1-0.5)/{\left(0.05\right)}^{\text{2}}\\ & =384 \end{array}$$

Where,

n = required sample size
z = 1.96 at 95% Confidence Interval (CI)
p = population proportion, 50%
q = 1-p
e = margin of error, 5%

The calculated sample size was 384. Since we used convenient sampling, we doubled the sample size to 768. Adding a nonresponse rate of 10%, the sample size became 844.8. However, 851 participants were enrolled in the study.

The data was collected using a performa consisting of demographic characteristics and histopathological variables. The data were coded, entered, and cleaned in Excel 2010 and analyzed using Statistical Package for Social Sciences (SPSS) version 20. Descriptive statistics were presented as frequency, percentage, mean, standard deviation using tables and figures. The study proposal was approved by the Institutional Review Committee (IRC) at BPKMCH (Ref: 247/2020) on 28th June 2020.

## RESULTS

**Figure 1.**
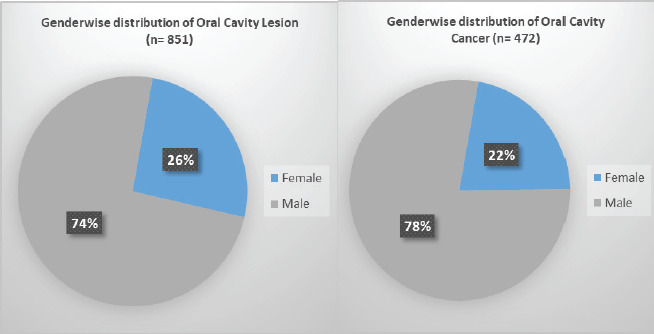
Gender-wise distribution of oral cavity lesion and cancer.

**Figure 2.**
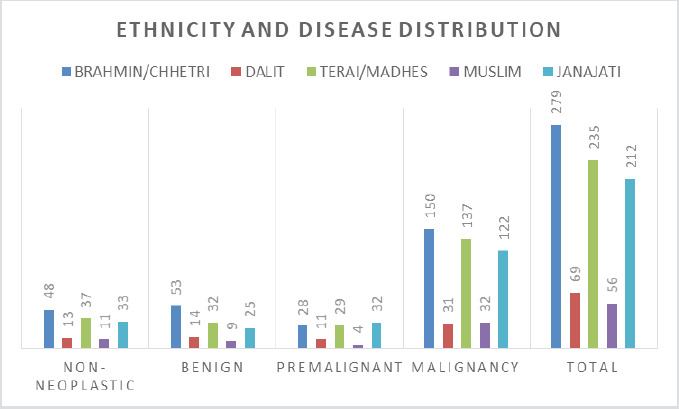
Ethnicity and disease distribution.

A total of 851 cases of oral cavity lesions were included in the study, with the mean age of the case 55.9 years. Three fourth of the cases were male (Figure 1). The most common age group was 46-75 years, 533 (62%) (Table 1). Ethnicity wise Brahmin/Chhetri has the highest incidence then followed by people from Terai/Madhes and then Janajati (Figure 2). Most of the lesions, 472 (55.5%) were malignant. No case of malignancy was less than 15 years.

**Table 1:** Age-wise oral lesion distribution.

Age group (in years)	Non-neo-plastic	Be-nign	Pre malignant	Malignant	n (%)
0-15	1	2	1	0	4 (0.5)
16-30	10	22	4	18	54 (6.3)
31-45	49	42	28	99	218 (25.6
46-60	41	48	41	182	312 (36.7)
61-75	33	16	27	145	221 (26.0)
>75	8	3	3	28	42 (4.9)
Total	142	133	104	472	851 (100.0)

**Figure 3.**
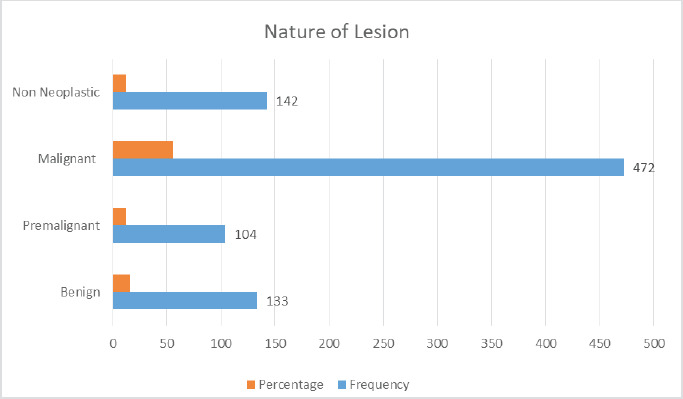
Distribution as per nature of the lesion.

More than half, 472 (55.5%) were malignant lesions with a mean age of presentation 55.9 years (range 20-93 years). Around 104 (12%) of oral cavity lesions were premalignant lesions, 133 (15%) were benign, and 142 (16%) were non-neoplastic lesions (Figure 3). Ninenty eight were dysplasia among premalignant conditions diagnosed (mild, moderate, and severe) . Five were lichen planus, and one was proliferative verrucous hyperplasia.

More than 453 (95%) malignant cases are squamous cell carcinoma, of which 342 (75%) were well-differentiated type, 92 (20.5%) moderately differentiated, and the remaining 19 (4.5%) were a poorly differentiated type. The rest of the cases were small round cell tumors (includes lymphoma, plasmacytoma, sarcoma), verrucous carcinoma (Table 2). One case of melanoma in the hard palate was diagnosed, which was a rare presentation location-wise. A case of basal cell carcinoma was diagnosed in the upper lip.

**Table 2:** Frequency and Types of Oral Cavity Cancer.

Histopathological findings	n (%)
Squamous Cell Carcinoma	453 (95.5)
Well Differentiated	342 (75.5)
Moderately Differentiated	92 (20)
Poorly differentiated	19 (4.5)
Mucoepidermoid Carcinoma	2 (0.4)
Basal Cell Carcinoma	1 (0.2)
Sarcoma	2 (0.4)
Carcinosarcoma	2 (0.4)
Small Round Cell Tumor	5 (1.2)
Adenoid Cystic Carcinoma	2 (0.4)
Verrucous Carcinoma	4 (1.0)
Melanoma	1 (0.4)

Around 212 (45%) malignant lesions occurred in the buccal mucosa, then 96 (20%) in the tongue, and 86 (18%) in the lower gingivobuccal region (Table 3). The location-wise tumor may involve multiple sites; buccal mucosa, sulcus, gingiva, and alveolus. However, the most involved site was considered.

**Table 3:** Site wise distribution.

Site	Oral cavity		Malignant	
	lesions	n (%)	lesions	n (%)
Buccal Mucosa	390	(45.8)	212	(44.9)
Floor of mouth	23	(2.7)	12	(2.6)
Lower Gingivobuccal Region	153	(17.9)	86	(18.2)
Hard Palate	21	(2.5)	16	(3.4)
Upper Gingivobuccal region	10	(1.2)	6	(1.3)
Lower lip	41	(4.8)	20	(4.2)
Retromolar trigone	22	(2.6)	8	(1.7)
Soft palate	18	(2.1)	15	(3.2)
Tongue	169	(19.9)	96	(20.3)
Upper lip	4	(0.6)	1	(0.2)
Total	851	(100.0)	472	(100.0)

## DISCUSSION

In this study, a total of 851 cases included; in a similar study, the number of the sample included were 21 cases by Pudasaini S et al.,^4^ 200 by Owais G et al.,^5^ 119 by Modi D et al.,^6^ 100 by Mehta N V et al.,^7^ 100 by Dolkahiya Z et al.,^8^ 1005 by Shyam N D et al.,^9^ 80 by Patro P et al.,^10^ which included a varying spectrum of pathology viz; Non-neoplastic, Benign, Premalignant and Malignant. Diagnosis of Malignant lesions in an early stage is of utmost importance for treatment with curative intent. As per BPKMCH annual report 2018^11^ and 2019,^12^ around 13% of cases are oral cavity cancer out of total cancer cases in the hospital.

Pre-malignant lesions and conditions like oral submucous fibrosis, leukoplakia, erythroplakia, and lichen planus also increase malignant transformation risk.^5^ In our study 12% of total cases presented in the premalignant status however 24% in Bhalekar H S et al,^13^ 19 % in Kosham S,^14^ 17% in Mehta N V,^7^ 43.3% in Owais G et al.^5^ Premalignant presentation is least in our study might be due to late seeking of healthcare and this being tertiary Onco Hospital, low referral of Premalignant cases. Although the oral cavity is more accessible to complete examination leading to early detection of pre-cancerous and cancerous lesions but might be due to ignorance or inaccessibility of medical care, the disease usually gets detected in later stages.^6,7^

Early stages of malignant lesions can also mimic benign lesions leading to incorrect diagnosis and treatment. In order to treat the patient, establishment of diagnosis is a must.^5,13^ Seventy-eight percent of cases are male, which is similar to the study done by Bhalekar H S et al.^13^ Pudasaini S et al.^4^ Globally, about 40% of men smoke as compared with nearly 9% of women. Compared to males, the use of tobacco products and alcohol is less in females, but a rising trend is seen recently.^15^

Factors considered to be associated with oral cancer are tobacco smoking, alcoholic consumption, betel quid chewing, poor oral health, and human papillomavirus infection.^3^ Distinct cultural practices such as betel-quid chewing and varying tobacco and alcohol use patterns among Asian Populations are considered to be predisposing factors for alarming increasing incidence rates. Alcohol can act as a local and systemic risk factor by increasing the oral mucosa's permeability, dissolving lipid components of the epithelium, causing epithelial atrophy and interference in DNA synthesis and repair; it has genotoxicity and mutagenic effects and also affects the liver's ability to clear chemical carcinogens.^4,16^

The present study showed a higher prevalence of oral mucosal lesions in the age group of 31 to 75 years (88 %), most probably caused due to the long-term use of tobacco and alcohol during this age period.

The majority of patients consumed tobacco in some form, which correlates with the fact that tobacco use is a known risk factor in developing oral cancer. The buccal mucosa is the commonest site of involvement (45%), similar to Modi D et al.^6^ and Wahi P N et al.^17^ Malignancy in the lower gingivobuccal sulcus and buccal mucosa is drastically high as chewing tobacco directly comes in contact with the same site signifying more prevalence of pan-chewing habits khaini, etc.

The most common malignancy was Squamous Cell carcinoma, which is concordance with the study done by Modi D et al.^6^ Patro P et al.^10^ Shyam N D et al.^9^ This study cannot be generalized as it is conducted in only one oncology center. However, BPKMCH is the largest comprehensive cancer care center in Nepal.

Out of squamous cell carcinoma (SCC), 75.5% was Well Differentiated Type, which is similar to 62.1% by Shyam N D et al.^9^ 53.8% by Prasan D.^18^ The youngest age of SCC was 20 years, and oldest 93 years was observed in our study. Mucoepidermoid and Adenoid Cystic Carcinoma are the salivary gland neoplasm observed in the oral cavity when arising from minor salivary glands.

Many diagnostic tests are used to detect oral cavity malignancies like vital staining, oral cytology, light-based detection, or oral spectroscopy. But histopathology is still the gold standard and most used technique for diagnosis.^19^

## CONCLUSIONS

This study reported that the most common oral cavity lesion was malignancy with a well-differentiated squamous cell carcinoma variant. Buccal mucosa involvement was the most common in oral cavity lesions and malignant lesions.
